# Intravitreal Anti-VEGF Injections Reduce Aqueous Outflow Facility in Patients With Neovascular Age-Related Macular Degeneration

**DOI:** 10.1167/iovs.16-20786

**Published:** 2017-03

**Authors:** Joanne C. Wen, Ester Reina-Torres, Joseph M. Sherwood, Pratap Challa, Katy C. Liu, Guorong Li, Jason Y. H. Chang, Scott W. Cousins, Stefanie G. Schuman, Priyatham S. Mettu, W. Daniel Stamer, Darryl R. Overby, R. Rand Allingham

**Affiliations:** 1 Department of Ophthalmology, University of Washington, Seattle, Washington, United States; 2 Department of Ophthalmology, Duke University Eye Center, Durham, North Carolina, United States; 3 Department of Bioengineering, Imperial College London, London, United Kingdom

**Keywords:** anti-VEGF, aqueous outflow facility, ocular hypertension, intraocular pressure

## Abstract

**Purpose:**

We assess the effect of intravitreal anti-VEGF injections on tonographic outflow facility.

**Methods:**

Patients with age-related macular degeneration who had received unilateral intravitreal anti-VEGF injections were recruited into two groups, those with ≤10 and those with ≥20 total anti-VEGF injections. Intraocular pressure and tonographic outflow facility of injected and uninjected fellow eyes were measured and compared between groups. Risk factors for development of reduced outflow facility also were assessed.

**Results:**

Outflow facility was 12% lower in the injected eyes of patients who received ≥20 anti-VEGF injections, compared to contralateral uninjected eyes (*P* = 0.02). In contrast, there was no facility reduction for patients with ≤10 anti-VEGF injections (*P* = 0.4). In patients with ocular hypertension in the uninjected eye (IOP > 21 mm Hg, *n* = 5), the outflow facility of injected eyes was on average 46% lower (*P* = 0.01) than in the uninjected fellow eyes. This was significantly greater than the difference observed in patients with IOP ≤ 21 mm Hg in the uninjected eye (*P* = 2 × 10^−4^). In patients with ocular hypertension in the injected eye (*n* = 6) the differences in facility and IOP between contralateral eyes were significantly greater than in patients with IOP ≤ 21 mm Hg in the injected eye (*P* = 2 × 10^−4^ and *P* = 7 × 10^−4^, respectively).

**Conclusions:**

Chronic anti-VEGF injections significantly reduce outflow facility in patients with AMD. The greatest facility reduction is observed in patients with baseline ocular hypertension. Ophthalmologists who administer anti-VEGF injections should be aware of these findings and monitor patients closely for changes in IOP or evidence of glaucoma, especially in those with pre-existing ocular hypertension.

Intravitreal anti-VEGF therapy, which includes bevacizumab (Avastin; Genentech, South San Francisco, CA, USA), ranibizumab (Lucentis; Genentech), and aflibercept (Eylea, VEGF-Trap Eye; Regeneron, Inc., Tarrytown, NY, USA) are used to treat a number of retinal vascular disorders, including neovascular age-related macular degeneration (NVAMD), diabetic macular edema, and retinal vein occlusion.^1–3^ Several million anti-VEGF injections are administered in the United States every year and this number continues to grow.^4^ Injection of anti-VEGF into the eye is associated with a transient, volume-dependent, elevation of IOP that typically resolves within 30 to 60 minutes.^5,6^ However, a number of observational studies have reported sustained IOP elevation, variably defined as IOP ≥ 21 or 22 mm Hg and elevation of ≥6 mm Hg from baseline on at least 2 consecutive visits, in 3% to 11% of patients receiving repeated anti-VEGF injections.^7–16^

The mechanism for sustained IOP elevation following intravitreal anti-VEGF is unknown. Proposed mechanisms include obstruction of the conventional outflow pathway by protein aggregates or foreign particles,^17^ or damage to the outflow pathway by local inflammation,^15^ mechanical trauma,^18^ chronic angle closure,^19^ or toxicity following repeated injections.^20^ Vascular endothelial growth factor may affect outflow facility^21^ and we show in our companion study that endogenous VEGF expression in the trabecular meshwork is a paracrine regulator of conventional outflow facility.^22^ Although these studies implicate reduced outflow facility as the mechanism for sustained elevated IOP in patients receiving chronic intravitreal anti-VEGF injections, to our knowledge, outflow facility has not been measured in patients receiving prolonged anti-VEGF injections.

The purpose of this study was to examine the effect of anti-VEGF therapy on measured outflow facility in patients with NVAMD. We compared the tonographic outflow facility between eyes of patients with unilateral NVAMD who have received longer and shorter courses of intravitreal anti-VEGF injections. We also sought to identify factors that may contribute to decreased tonographic outflow facility including pre-existing ocular hypertension.

## Methods

### Patient Enrollment

This study followed the tenets of the Declaration of Helsinki and was approved by the Duke University Institutional Review Board. All subjects gave written informed consent before study inclusion. Patients aged >50 years with a history of monocular NVAMD treated with intravitreal anti-VEGF injections were recruited from the Duke Eye Center Retina Clinics. Since mean values of outflow facility and IOP are not significantly different between paired eyes of normal (nonglaucomatous) individuals,^23,24^ we recruited patients receiving unilateral anti-VEGF injections to compare injected versus fellow uninjected eyes. In this manner each patient served as his or her own control for analyses. Patients were recruited into two groups: a low injection (≤10 injections) and high injection (≥20 injections) group. Exclusion criteria were the same for injected and uninjected fellow eyes and are listed in Table 1.

**Table 1:**
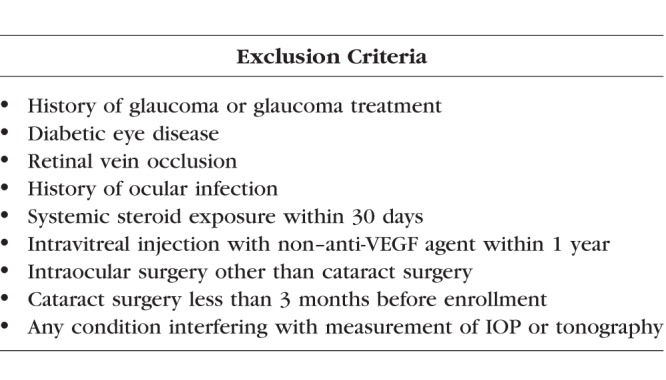
Exclusion Criteria for Injected and Uninjected Fellow Eyes

Demographic information, including patient age, sex, ethnicity, ocular and medical history, ocular medications, and number and type of intravitreal anti-VEGF injections, was collected.

### Outflow Facility Measurements

Intraocular pressure and tonographic outflow facility measurements were performed before a scheduled injection and at least 4 weeks after the last intravitreal injection. An average of 3 IOP measurements per eye were obtained using an iCare Rebound (Tiolat Oy, Helsinki, Finland) tonometer without topical anesthesia. The patient then was placed in the supine position and topical proparacaine was administered to both eyes. The outflow facility of each eye was measured by a single investigator (JCW) using an electronic Schiøtz tonograph (V-Mueller and Co., Chicago, IL, USA). A 4-minute tracing was recorded using a 5.5 g weight. The outflow facility was calculated using standard interpretation methods where a best fit line along the tonographic tracing was assigned to determine the starting and ending points. All tracings were measured in a masked fashion by one investigator (RRA). Tonography tracings with significant irregularities from patient blinking or eye movement, or tracings without ocular pulsations for >30 seconds were considered technically unsatisfactory and excluded from analysis.

### Statistical Analysis

Demographic data for low and high injection groups were compared using a χ^2^ square analysis. Differences between IOP and tonographic outflow facility in contralateral eyes were compared using paired *t*-tests. Unpaired *t*-tests were used to analyze the differences in outflow facility and IOP between groups. A *P* value <0.05 was considered statistically significant.

The sample size required to detect a 15% difference in tonographic outflow facility between injected and uninjected eyes in each group was calculated. Assuming a mean outflow facility of 0.23 ± 0.05 μL/min/mm Hg reported for our older patient demographic,^25,26^ it was estimated that 20 patients would provide an 80% chance to detect a 15% difference in the outflow facilities for each group (paired *t*-test, *α* = 0.05, *β* = 0.20).

## Results

Of 46 patients recruited for this study, 22 were in the low injection group and 24 in the high injection group. All patients had unilateral injections of bevacizumab (*n* = 2), ranibizumab (*n* = 1), aflibercept (*n* = 11), or a combination (*n* = 32). Four patients were excluded from the analysis (2 in the ≤10 and 2 in the ≥20 injection groups) due to poor quality tonographic tracings. Therefore, 20 and 22 patients were included in the final analysis for the low and high injection groups, respectively.

The baseline patient characteristics of the 2 groups are shown in Table 2. The patient demographics did not differ significantly between the 2 groups. As expected, the mean number of injections between groups was significantly different (6.3 ± 3.2 injections (range, 1–10) for the low injection group versus 30.3 ± 13.3 injections (range, 20–68) for the high injection group; *P* < 0.001). Average values of facility and IOP for each of the groupings are provided in Table 3.

**Table 2:**
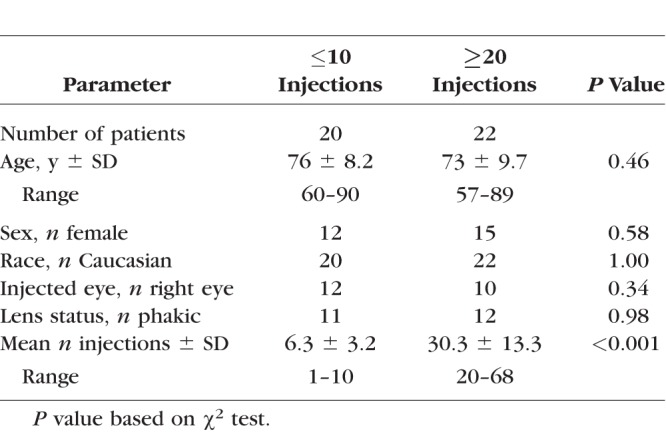
Baseline Demographics of Patients With ≤10 and ≥20 Injections

**Table 3:**
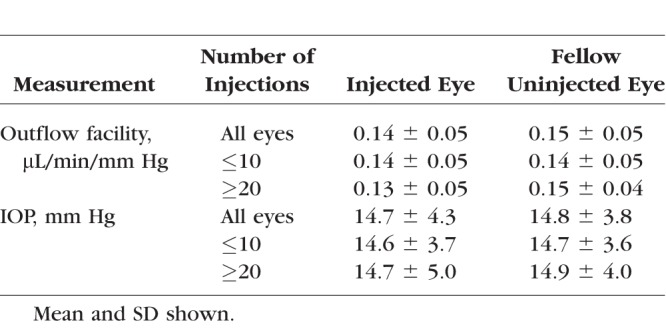
Comparison of Outflow Facilities and IOP Between Subjects With ≤10 and ≥20 Anti-VEGF Injections

**Figure 1:**
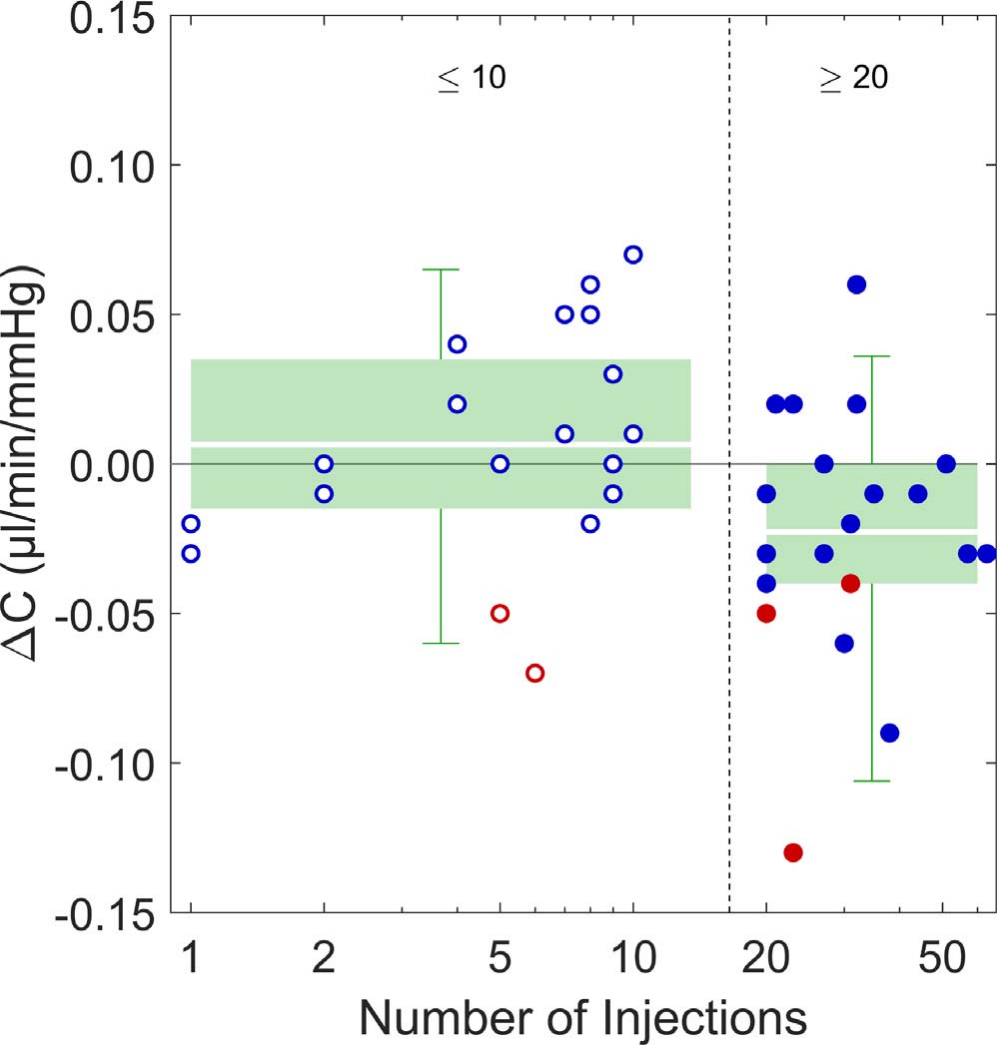
Difference in outflow facility (ΔC) between injected eyes and fellow uninjected eyes, grouped by ≤10 injections (n = 20, hollow circles) and ≥20 injections (n = 22, filled circles). Blue data points indicate patients who were normotensive in the uninjected eye (IOP < 21 mm Hg); red data points indicate patients who were ocular hypertensive in the uninjected eye (IOP ≥ 21 mm Hg) in the uninjected eye. Green boxes indicate the inner interquartile range, error bars indicate the range encompassing 95% of measured values, while white lines through the boxes represent the mean values. ΔC < 0 indicates a lower outflow facility in the injected eye.

The pairwise differences in outflow facility between injected and uninjected fellow eyes (ΔC) in the high injection group (≥20 injections), were ΔC = −0.02 ± 0.04 μL/min/mm Hg (Fig. 1), corresponding to a difference of 12 ± 27% relative to the uninjected eye (*P* = 0.01). In contrast, there was no difference in tonographic outflow facility in eyes with ≤10 injections compared to their fellow eyes (ΔC = 0.01 ± 0.04 μL/min/mm Hg, *P* = 0.4). The average difference in ΔC for the high injection group was significantly different from that in the low injection group (*P* = 0.02). There was no significant correlation between total number of injections and the difference in outflow facility between paired eyes (*r* = −0.23, *P* = 0.14).

**Figure 2:**
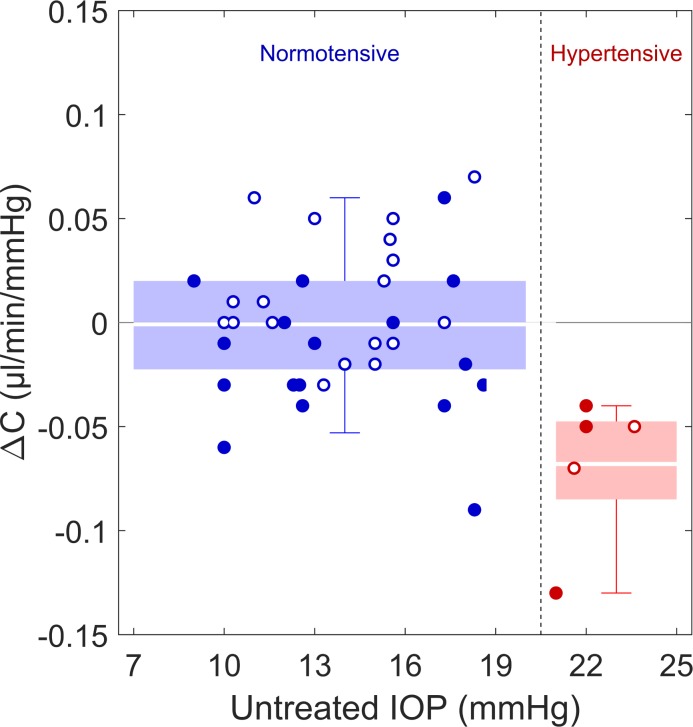
Difference in outflow facility (ΔC) between injected eyes and fellow uninjected eyes, grouped by IOP of the uninjected eye (blue, normotensive ≤ 21 mm Hg; red, hypertensive > 21 mm Hg). Hollow circles received ≤ 10 injections and filled circles received ≥ 20 injections. Box plots defined as in Figure 1.

To assess whether patients exhibiting baseline ocular hypertension independent of anti-VEGF therapy are more susceptible to a reduction in outflow facility in response to anti-VEGF, patients were divided based on IOP of the uninjected eye into an ocular hypertensive group (IOP >21 mm Hg, *n* = 5) and a normotensive group (IOP ≤ 21 mm Hg, *n* = 37). The IOP of the uninjected eye was used to reflect the status of the injected eye before anti-VEGF treatment. The mean outflow facility in injected eyes was on average 46% lower than that in uninjected fellow eyes for the group of ocular hypertensive cases (0.08 ± 0.02 vs. 0.15 ± 0.04 μL/min/mm Hg respectively, ΔC = −0.07 ± 0.03 μL/min/mm Hg; *P* = 0.01, Fig. 2). In contrast, the average facility for the normotensive group was not different for injected versus uninjected fellow eyes (0.14 ± 0.04 vs. 0.15 ± 0.05 μL/min/mm Hg, respectively; ΔC = 0.00 ± 0.03 μL/min/mm Hg; *P* = 0.9). The difference in the average ΔC between the ocular hypertensive and normotensive groups was highly significant (*P* = 2 × 10^−4^). The difference in facility between injected and uninjected eyes for the ocular hypertensive group, however, did not coincide with a difference in IOP (ΔP = −0.7 ± 3.5 mm Hg, *P* = 0.66). Similarly, no difference in IOP between injected and uninjected eyes was observed for the normotensive group (ΔP = −0.1 ± 2.4 mm Hg, *P* = 0.88). This suggests that a reduction in outflow facility in an otherwise healthy eye is not necessarily sufficient to induce ocular hypertension, possibly due to homeostatic mechanisms or secondary effects of anti-VEGF that act on other aspects of aqueous humor dynamics.

Patients who are ocular hypertensive in the injected eye (IOP > 21 mm Hg) are at risk of glaucomatous damage due to anti-VEGF therapy. In all of these patients (*n* = 6), IOP was greater in the injected eye compared to the contralateral uninjected eye (ΔP = 3.2 ± 2.4 mm Hg, *P* = 0.021), indicating that regardless of whether these patients were originally ocular hypertensive, anti-VEGF treatment led to a further elevation in IOP. The mean outflow facility in these injected eyes also was significantly lower than in the uninjected fellow eyes (0.095 ± 0.05 vs. 0.15 ± 0.05 μL/min/mm Hg, respectively, ΔC = −0.06 ± 0.04 μL/min/mm Hg, *P* = 0.02).

**Figure 3:**
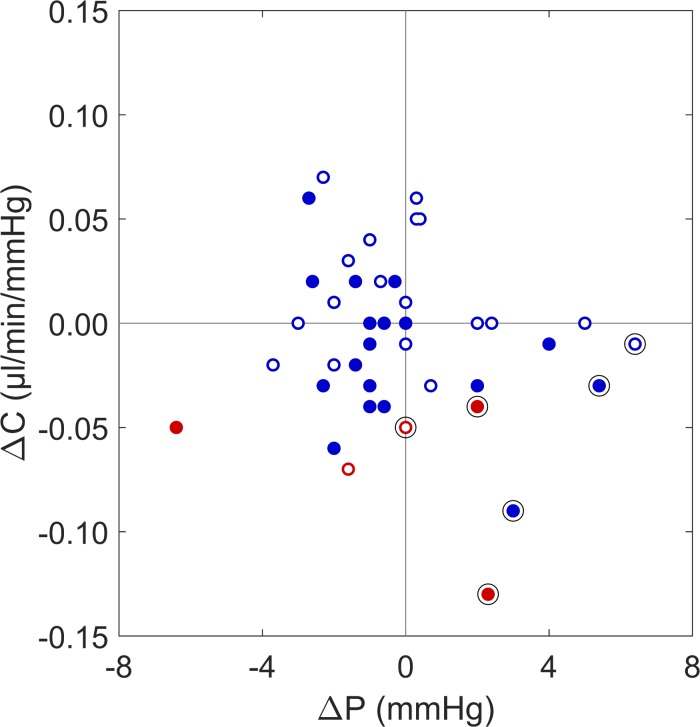
Difference in outflow facility (ΔC) versus difference in IOP (ΔP) between injected eyes and fellow uninjected eyes. Color schemes follow Figures 1 and 2. ΔC < 0 indicates a lower outflow facility in the injected eye and a ΔP > 0 indicates a higher IOP in the injected eye. Cases of ocular hypertension in the injected eye are indicated by circled data points and all such cases demonstrate lower outflow facility and higher IOP compared to the fellow uninjected eye.

As shown in Figure 3, all cases of ocular hypertension in the injected eye (indicated by circled data points) fell in the lower right quadrant, indicating that these individuals exhibited elevated IOP and decreased facility in the injected eye, relative to the fellow uninjected eye. In contrast, cases without ocular hypertension in the injected eye were distributed throughout all four quadrants and did not exhibit either a difference in facility (ΔC = 0.00 ± 0.03 μL/min/mm Hg, *P* = 0.9) or a difference in IOP (ΔP = −0.7 ± 2.1 mm Hg, *P* = 0.05) between injected and uninjected eyes. The average values of ΔC and ΔP in those with ocular hypertension in the injected eye were significantly different from those without ocular hypertension in the injected eye (*P* = 7 × 10^−4^ for ΔC and *P* = 2 × 10^−4^ for ΔP). These data demonstrated that, for the subpopulation of patients who were ocular hypertensive following anti-VEGF therapy, increased IOP coincided with a reduction in outflow facility.

## Discussion

To our knowledge, this is the first study to examine the effect of chronic intravitreal anti-VEGF therapy treatment on conventional outflow facility (PubMed [MEDLINE] search terms: outflow, anti-VEGF). We found a small but statistically significant decrease in tonographic outflow facility in the patients with a high number of anti-VEGF injections (≥20 injections) when comparing injected eyes with their uninjected fellow eyes. This was not observed in patients with fewer (≤10) total injections. More significantly, we found that patients with ocular hypertension in their uninjected eye demonstrated a substantially lower outflow facility (46% decrease) in their injected eye.

The original clinical trials that examined the efficacy of intravitreal anti-VEGF injections in the treatment of NVAMD did not report significant changes in IOP following treatment.^1–3^ However, several reports, including a post hoc analysis of the MARINA and ANCHOR trials,^27^ have since described cases of sustained IOP elevation following intravitreal anti-VEGF injections.^7–16^ These studies hypothesize that the IOP elevation is due to an impairment of outflow facility; however, none of these clinical trials studied the effect of anti-VEGF therapy on aqueous outflow facility.

In the present study, two analyses were performed to investigate the hypotheses that: (1) prolonged exposure to anti-VEGF therapy is a risk factor for a reduction in outflow facility, and (2) patients with evidence for impaired outflow facility, in this case ocular hypertension, were more likely to experience a substantial reduction in outflow facility from anti-VEGF treatment. In the first analysis, we found a moderate reduction of outflow facility in eyes with a high number of injections (≥20) compared to their uninjected fellow eyes. These findings suggested that increasing numbers of anti-VEGF injections are associated with a modest decline in outflow facility on average. In the second analysis, we compared patients with baseline ocular hypertension in the uninjected fellow eye (IOP > 21 mm Hg) to the normotensive group (IOP ≤ 21 mm Hg), with the rationale being that the uninjected eye provides a reasonable reflection of the baseline status of the injected eye before anti-VEGF treatment. In patients with baseline ocular hypertension, the injected eye consistently demonstrated almost a 2-fold reduction in outflow facility compared to the uninjected eye. From these data, we concluded that ocular hypertension is an additional risk factor for facility reduction in response to anti-VEGF therapy. Interestingly, of the 5 ocular hypertensive patients, 2 had received ≤10 total injections and yet already demonstrated reduced outflow facility in the injected versus fellow eyes. These findings suggested that baseline ocular hypertension, therefore, may be a greater risk factor for development of impaired outflow facility than number of anti-VEGF injections.

This result also may explain why only a subset of patients receiving intravitreal anti-VEGF injections exhibit elevated IOP following chronic exposure. The most dramatic differences in outflow facility among paired eyes were observed in subjects who had ocular hypertension, defined by IOP > 21 mm Hg, in either the injected or uninjected eye. This suggested that the patients who are most at risk appear to be those who start anti-VEGF treatment with an unhealthy trabecular meshwork, which typically is associated with ocular hypertension. Because the trabecular meshwork already may be stressed in these patients, the additional burden of anti-VEGF therapy increases the risk for further IOP elevation. This burden is imposed, presumably, by anti-VEGF compounds disrupting endogenous VEGF signaling involved in regulating outflow through the trabecular meshwork, as described in our companion study.^22^ Therefore, it may be prudent to establish the baseline IOP before starting intravitreal anti-VEGF injections, to identify those patients who may be at higher risk for these adverse consequences.

Our findings were consistent with the retrospective studies examining the incidence of sustained IOP elevation following anti-VEGF injections. Most studies reported an incidence of approximately 3% to 11% for development of sustained IOP elevation after anti-VEGF injection.^16^ In an analysis by Hoang et al.,^11^ the risk for sustained elevation of IOP was greatest in patients with ≥29 injections compared to patients with ≤12 injections (odds ratio [OR], 16.1; *P* = 0.008). Similarly, we observed a significant decline in aqueous outflow facility in patients with ≥20 injections where the mean number was approximately 30 injections. In the two studies that did not report an increased incidence of sustained IOP elevation following anti-VEGF injection, the mean number of injections was 8.4 and 9.5.^28,29^ This also is consistent with our findings that outflow facility is not significantly decreased in patients with ≤10 total injections. These studies paralleled our own observations regarding reduced outflow facility by suggesting that, in the case of sustained IOP, increased number of injections is associated with greater risk of an adverse event.

The exact mechanism of outflow reduction remains unknown. Our companion study (VEGF as a Paracrine Regulator of Conventional Outflow Facility) demonstrates that endogenous VEGF expression in the trabecular meshwork is a paracrine regulator of conventional outflow facility, which may be perturbed by the anti-VEGF agents themselves.^22^ Other studies have suggested that a number of potential mechanisms may contribute to IOP elevation, which include inflammation, obstruction by particulates in the injectate, or by secondary angle closure.^17,19^ Because the patients in this study were being treated for AMD and not glaucoma, gonioscopy was not performed to evaluate the status of angle structures. This would be helpful in future studies to better characterize mechanisms contributing to development of ocular hypertension in this patient population. Lastly, whether this decrease in outflow facility changes following cessation of anti-VEGF treatment is unknown. The vast majority of patients in this study received anti-VEGF injections on a regular schedule, typically every 4 to 6 weeks. There were too few patients with longer injection intervals to analyze the effect of injection spacing on outflow facility.

A limitation of our study is the lack of preinjection, baseline data on a number of our subjects. Many age-related macular degeneration (AMD) patients had received bilateral anti-VEGF therapy, so they were excluded from the study. In those who met the unilateral criterion, information on baseline IOP was limited or not available because treatment was initiated at other institutions. For this reason, we used the IOP of the uninjected eye as an estimate of baseline, preinjection status of both eyes. Therefore, an IOP of >21 mm Hg in the uninjected eye was assumed to represent a baseline, preinjection status of ocular hypertension. A number of studies report monocular treatment trials of glaucoma medications to assess efficacy, where the untreated eye is used as a control for comparison.^30,31^ However, anti-VEGF is known to exit the eye via the conventional outflow tracts and can be measured systemically following intravitreal injection.^32,33^ Therefore, unilateral injections still could have effects on the contralateral eye. In fact, studies in diabetic macular edema have reported that injection into one eye is associated with improved macular edema in the fellow uninjected eye.^34^ Yet, even if the anti-VEGF medication affected the uninjected eye, the magnitude of the effect on outflow facility would be expected to be the same or less. Therefore, if the fellow uninjected eye were exposed to anti-VEGF, it would likely reduce rather than increase the asymmetry between paired eyes, making this analysis more conservative. Regardless, prospective, longitudinal studies with complete preinjection and follow-up data will be needed to corroborate the findings from this study.

The mean outflow facilities in our study were lower than expected compared to other published reports for older patients that had a mean age of approximately 60 years.^25,26^ However, tonographic outflow facility is known to decrease with age.^25^ The mean age of patients in our study was approximately 15 years greater than the mean age of patients in the prior studies that were referenced.^25,26^

A number of studies have reported on the IOP lowering effects of cataract surgery and some suggest that this is due to an increase in outflow facility.^35–37^ This could potentially confound the facility comparisons in subjects with pseudophakia, especially if only one eye is pseudophakic. However, only 4 subjects were unilaterally pseudophakic. The remaining subjects were either bilaterally phakic or bilaterally pseudophakic. In all four unilaterally pseudophakic patients, the pseudophakic eye also was the injected eye. Assuming outflow facility was increased following cataract surgery, the effect of pseudophakia in the injected eye would tend to decrease the difference in outflow facility between injected and uninjected eyes, and, therefore, oppose the observed reduction of outflow facility in eyes receiving injections. To further address this important issue, we performed an analysis limited to subjects who were bilaterally phakic. In the low injection group, there still was no significant difference in outflow facility between eyes (ΔC = 0.00 ± 0.04 μL/min/mm Hg; *P* = 0.73, *n* = 11), and in the high injection group, outflow facility in the injected eyes was significantly lower on average than in the contralateral, uninjected eyes (ΔC = −0.03 ± 0.04 μL/min/mm Hg; *P* = 0.02, *n* = 12). Therefore, it appears unlikely that lens status accounts for effects of anti-VEGF injections on outflow facility.

We excluded patients with glaucoma who were or had been treated with IOP lowering medications or therapy to assess the effect of anti-VEGF therapy in isolation and to avoid the confounding effect of IOP lowering therapy on this study. However, these data do raise the possibility that chronic anti-VEGF therapy might pose special risks to glaucoma patients. Of note, pre-existing glaucoma and a family history of glaucoma have been reported as risk factors for development of sustained elevations of IOP after anti-VEGF injections.^10,11^ Ultimately, a more highly powered, longitudinal prospective study that includes glaucoma suspects and those with glaucoma will be needed to fully characterize the relationship between anti-VEGF therapy and aqueous outflow facility in this at risk patient population.

In conclusion, our data suggest that conventional outflow facility is reduced in response to anti-VEGF therapy. This effect may have a contributing role in cases where anti-VEGF therapy has been associated with sustained elevation of IOP. We also observed that patients with baseline ocular hypertension had greater reduction in outflow facility. Anti-VEGF injections are widely and increasingly used for AMD and other retinal vascular disorders. Chronic exposure of anti-VEGF therapy to this large number of patients, including those with ocular hypertension and glaucoma, may have significant clinical impact, including risk of glaucomatous visual field loss in a population that has experienced or is at risk for retinal disease. These data suggest that ophthalmologists who administer anti-VEGF injections should be aware of these findings and monitor patients closely for changes in IOP or evidence of glaucoma, especially in those with pre-existing ocular hypertension. Larger, prospective studies are needed to better understand the consequences of chronic exposure of anti-VEGF therapy.
